# Curative Effects of Thiacremonone against Acetaminophen-Induced Acute Hepatic Failure via Inhibition of Proinflammatory Cytokines Production and Infiltration of Cytotoxic Immune Cells and Kupffer Cells

**DOI:** 10.1155/2013/974794

**Published:** 2013-07-11

**Authors:** Yu Ri Kim, Nam Jin Lee, Jung Ok Ban, Hwan Soo Yoo, Yong Moon Lee, Yeo Pyo Yoon, So Young Eum, Heon Sang Jeong, Do-young Yoon, Sang Bae Han, Jin Tae Hong

**Affiliations:** ^1^College of Pharmacy and Medical Research Center, Chungbuk National University, 12 Gaeshin, Heungduk, Cheongju, Chungbuk 361-763, Republic of Korea; ^2^Agriculture, Life and Environments Sciences, Chungbuk National University, 12 Gaeshin, Heungduk, Cheongju, Chungbuk 361-763, Republic of Korea; ^3^Laboratory of Cytokine Immunology, Institute of Biomedical Science and Technology, College of Medicine, Konkuk University, Seoul 143-701, Republic of Korea; ^4^College of Pharmacy, Chungbuk National University, 48 Gaesin-dong, Heungduk-gu, Cheongju, Chungbuk 361-763, Republic of Korea

## Abstract

High doses of acetaminophen (APAP; *N*-acetyl-*p*-aminophenol) cause severe hepatotoxicity after metabolic activation by cytochrome P450 2E1. This study was undertaken to examine the preventive effects of thiacremonone, a compound extracted from garlic, on APAP-induced acute hepatic failure in male C57BL/6J. Mice received with 500 mg/kg APAP after a 7-day pretreatment with thiacremonone (10–50 mg/kg). Thiacremonone inhibited the APAP-induced serum ALT and AST levels in a dose-dependent manner, and markedly reduced the restricted area of necrosis and inflammation by administration of APAP. Thiacremonone also inhibited the APAP-induced depletion of intracellular GSH, induction of nitric oxide, and lipid peroxidation as well as expression of P450 2E1. After APAP injection, the numbers of Kupffer cells, natural killer cells, and cytotoxic T cells were elevated, but the elevated cell numbers in the liver were reduced in thiacremonone pretreated mice. The expression levels of I-309, M-CSF, MIG, MIP-1**α**, MIP-1**β**, IL-7, and IL-17 were increased by APAP treatment, which were inhibited in thiacremonone pretreated mice. These data indicate that thiacremonone could be a useful agent for the treatment of drug-induced hepatic failure and that the reduction of cytotoxic immune cells as well as proinflammatory cytokine production may be critical for the prevention of APAP-induced acute liver toxicity.

## 1. Introduction

Acetaminophen (APAP; *N*-acetyl-*p*-aminophenol) is a commonly used analgesic and antipyretic drug that is regarded as being safe at therapeutic doses. However, when taken in excess, it produces severe hepatotoxicity, which is termed centrilobular hepatic necrosis [1]. APAP is metabolically activated by cytochrome P450 2E1 to form a reactive metabolite, *N*-acetyl-p-benzoquinone imine (NAPQI), that covalently binds to proteins [2]. At therapeutic dosages, NAPQI is efficiently detoxified by glutathione (GSH) to form an APAP-GSH conjugate that is excreted by the kidney [3]. However, in APAP overdose, the sulfate and glucuronide conjugation pathways become saturated and the amount and the rate of formation of NAPQI are greatly increased [4]. NAPQI covalently binds to hepatic cellular proteins to form 3-(cysteine-S-yl)-acetaminophen (APAP-Cys) adducts [1, 5, 6]. Excessive NAPQI also peroxynitrites protein and oxidizes macromolecules such as lipid, protein, and DNA causing hepatic cellular injury and necrosis [7, 8]. 

Kupffer cells, natural killer (NK) cells, neutrophils, and macrophages contribute to APAP-induced hepatotoxicity through the release of proinflammatory cytokines and mediators, including tumor necrosis factor-alpha (TNF-*α*), interferon-gamma (IFN-**γ**), interleukin-(IL-) 1*α*, IL-1*β*, and nitric oxide (NO) [9, 10]. In APAP-treated mice, overproduction of IFN-**γ** significantly induces inflammatory cytokines, chemokines, adhesion molecules, Fas and inducible nitric oxide synthase (iNOS) [11, 12]. Overexpression of IFN-**γ** increases necrotic hepatotoxicity as indicated by serum alanine aminotransferase (ALT) and aspartate transaminase (AST) levels and by histopathological evaluation of necrosis [11, 13]. Inhibition of TNF-*α* or IL-1*α* with specific antibodies bestows protection against APAP-induced liver injury in mice [14]. IL-1ra is structurally similar to IL-1, which can bind solidly to the IL-1 receptor and block the IL-1 signal pathway [15]. The level of intrahepatic IL-1 is correlated with the severity of APAP-induced liver injury [13]. Activated Kupffer cells are important in NO and superoxide production, which leads to peroxynitrite formation [7, 8]. Kupffer cells may also counteract inflammation or have a role in liver repair through changes of IL-10 release [16]. Infiltrated NK and NK-T cells activated by Kupffer cells are the primary cell types in IFN-*γ* production in acetaminophen toxicity [11, 12]. IL-1 and IL-1ra are produced abundantly by various kinds of cells such as neutrophils, macrophages, and fibroblasts in patients with inflammatory conditions [15]. 

When the defense mechanisms are not sufficient to withstand the damaging attacks, cells start to synthesize chemokines such as monokine induced by gamma interferon (MIG) [17], gamma-interferon-inducible protein (IP-10) [17], cytokine-induced neutrophil chemoattractant (KC), and macrophage inflammatory proteins (MIPs) including MIP-1, MIP-2, and MIP-3 [18] that are thought to be responsible for attracting inflammatory cells like granulocytes and mononuclear phagocytes and for activating resident macrophages [19]. These chemokines are secreted by damaged hepatocytes, T, NK, and Kupffer cells in the acute inflammatory state [11, 12, 20]. On the other hand, IP-10 (CXCL10) and MIP-2 (CXCL-2) may lead to increased nuclear localization of the transcription factor signal transducer and the activator of transcription 3 (STAT3), a major signal transduction factor in hepatocyte regeneration [21–23]. In particular, IP-10 may be able to induce hepatocyte growth factor (HGF) [6] and protect the MIP-2 receptor against APAP hepatotoxicity [24]. Thus, increased production of MIP-2 and IP-10 is important in cell proliferation in response to APAP toxicity [6, 24].

Nuclear transcription factor-*κ*B (NF-*κ*B) is a transcription factor that regulates various genes involved in the production of inflammatory cytokines, chemokines, cell adhesion molecules, and growth factors [25]. NF-*κ*B also regulates the expression of a number of antiapoptotic proteins as well as proliferation genes [26]. Following an overdose of APAP, the NF-*κ*B DNA binding activity in the liver is increased, and the protective effects against APAP-induced toxicity are associated with reduced NF-*κ*B [27]. STAT1 has also been implicated in hepatic inflammation and injury as well as suppression of liver regeneration [27]. STAT-1 is activated in response to IFN-*γ* in concanavalin-A induced hepatitis and the lipopolysaccharide/d-galactosamine-induced hepatitis model and plays a harmful role in these models of hepatotoxicity [28, 29]. On the other hand, STAT3, which is mainly activated by IL-6 and its related cytokine, has key roles in acute phase responses that include protecting against liver injury and promoting liver regeneration [30–32]. Disruption of the STAT3 gene impairs liver regeneration and activated STAT3 ameliorates fatty liver disease [30–32]. These findings suggest that STAT3 plays an important role in hepatoprotection and liver regeneration by inducing a variety of antiapoptotic and mitogenic proteins, whereas STAT1 mediates hepatic injury.

Garlic has been used in traditional medicine as a food component for the treatment of cancer, obesity, diabetes, hepatotoxicity, nephrotoxicity, and cardiovascular diseases through the modification of risk factors like hypertension, high blood cholesterol, and thrombosis, and by acting on other acute and chronic diseases associated with oxidative stress and aging [33–35]. These pharmacological effects of garlic are attributed to the presence of pharmacologically active sulfur compounds including diallyl sulfide, diallyl disulfide, allicin and dipropyl sulfide [36, 37]. These compounds increase the activity of enzymes involved in the metabolism of carcinogens [38] along with their anti-oxidative activities [39] and anti-inflammatory effects *in vitro* and *in vivo* [40–42]. 

We previously isolated a novel sulfur compound from garlic and identified it as thiacremonone (2,4-dihydroxy-2,5-dimethylthiophene-3-one); subsequent studies revealed its antiobesity effects through suppression of peroxisome proliferator-activated receptor-gamma (PPAR-*γ*) transcriptional activity via the activation of AMP-activated protein kinase [43]. Moreover, thiacremonone was shown to have anti-inflammatory effects through the inhibition of the NF-*κ*B pathway [36, 37, 44]. In this study, we investigated the protective role of thiacremonone in APAP-induced hepatotoxicity and investigated the involvement of NF-*κ*B and STAT1 pathways in the action of thiacremonone during acute hepatic failure.

## 2. Methods and Materials 

### 2.1. Ethics Statement

All animal experiments were approved and carried out according to the Guide for the Care and Use of Animals, Animal Care Committee of Chungbuk National University (CBNUA-144-1001-01).

### 2.2. Animals and Treatments

C57BL6/J male mice were purchased from Orient Bio Inc. (Seongnam-si, Gyeonggi-do, Republic of Korea). The mice were housed and bred under specific pathogen-free conditions at the Laboratory Animal Research Center of Chungbuk National University, Republic of Korea. The mice (*n* = 4/cage) were maintained in a room with a constant temperature of 22 ± 3°C and a relative humidity of 50 ± 10% under a 12-h light/dark cycle, and were fed standard rodent chow (Samyang, Korea) with purified tap water *ad libitum*. Mice matched for age (9 weeks old) and weight (18–24 g) (*n* = 12/group) were used. Before injection of APAP, the mice were intraperitoneally (i.p.) administered thiacremonone (purity >97%, 10, 20, or 50 mg/kg dissolved in phosphate buffered saline, PBS) once daily for 7 days. Thiacremonone was isolated and characterized as previously described [45–47]. APAP was dissolved in saline by heating at 60°C. The mice were fasted for 16 h and received the i.p. injection of APAP (500 mg/kg; Sigma-Aldrich, St. Louis, MO) or saline. The mice were sacrificed 40 h after APAP injection. The mice were observed during the 40 h period to calculate the overall survival.

### 2.3. Serum AST and ALT Measurements

Mice were anesthetized with an overdose of pentobarbital (100 mg/kg) and blood was taken by heart puncture. Serum levels of ALT and AST were measured using individual assay kits (Sigma Diagnostics, St. Louis, MO).

### 2.4. Hepatic Glutathione/Oxidized Glutathione Ratio and Nitric Oxide Measurements

Hepatic levels of reduced glutathione (GSH) and oxidized glutathione (GSSG) were measured using a commercial assay kit (Cayman Chemical, Ann Arbor, MI). Briefly, frozen tissues were placed into the buffer at a ratio of 10% (tissue weight/volume of buffer). Tissue homogenates were prepared in 0.1 M phosphate buffer (pH 7.4) containing ethylenediaminetetraacetic acid (EDTA) either supplemented with 0.01 M N-ethylmaleimide for assessing GSSG or without N-ethylmaleimide for assessing GSH. Tissue contents of both GSH and GSSG were measured by the enzyme recycling method as previously described [48]. Liver tissues were homogenized in ice-cold lysis buffer (pH 7.5, 50 mM Tris, 1% Nonidet P-40, 150 mM NaCl, and a cocktail of proteinase inhibitors) and were centrifuged at 2000 g for 20 min. The supernatant was kept at −80°C until use. Nitric oxide (NO) concentration was determined by indirect measurement of nitrite, a byproduct of NO transformation in living tissues. An NO detection kit was used to analyze the concentration of NO (iNtRON Biotechnology, Seoul, Republic of Korea), and the assay was performed as described in the manufacturer's protocol.

### 2.5. Western Blotting

Liver tissues were homogenized with protein extraction solution (PRO-PREP, iNtRON Biotechnology) and were lysed prior to 60 min incubation at −20°C. The tissue lysate was centrifuged at 15,000 rpm for 15 min at 4°C. Equal amounts of proteins (30 *μ*g) were separated by 10% sodium dodecyl sulfate-polyacrylamide gel electrophoresis and transferred to a polyvinylidene difluoride (PVDF) membrane (GE Water and Process Technologies, Trevose, PA). Blots were blocked for 1 h at room temperature with 5% (w/v) skim milk in Tris-Buffered Saline Tween-20 (TBST: 10 mM Tris (pH 8.0) and 150 mM NaCl solution containing 0.05% Tween-20). After a short wash in TBST, the membranes were immunoblotted with the following primary antibodies; rabbit polyclonal antibodies directed against cytochrome P450 2E1 (1 : 2000 dilution; Abcam PLC, Cambridge, MA), p65 and p50 (1 : 2000 dilution; Santa Cruz Biotechnology, Santa Cruz, CA), and STAT1 or p-STAT1 (1 : 2500 dilutions; Santa Cruz Biotechnology). Each blot was incubated with the respective horseradish peroxidase-conjugated anti-rabbit or anti-mouse IgG (1 : 4000 dilution; Santa Cruz Biotechnology). Immunoreactive proteins were detected with the ECL detection system.

### 2.6. Cytokine Immuno-Arrays

Liver tissues were excised and homogenized in PBS with protease inhibitor cocktail (Sigma-Aldrich) and Triton X-100 (final concentration 1%). The samples were frozen at −70°C, thawed, and centrifuged at 10,000 ×g for 5 min to remove cellular debris. The tissue lysates were used to perform protein array in the same way as for Western blotting. Protein (4.5 mg) collected from three samples per group was used for Proteome Profiler mouse cytokine array as per the protocol provided by the supplier (ARY006; R&D Systems, Minneapolis, MN). Briefly, the antibody-printed membrane was blocked for 1 h at 23°C. Liver lysate (0.5 mL) was mixed with biotinylated antibody cocktail and incubated for 1 h at 23°C. Next, the mixture was added to the membrane and incubated overnight at 4°C. After multiple washing, the membrane was incubated in streptavidin-horseradish peroxidase solution for 30 min at room temperature of 23°C. The immunocomplex was detected by using ECL.

### 2.7. Flow Cytometry Analysis

Immune-related cells were obtained from the liver for phenotype analysis. Liver tissues were isolated from fresh liver biopsies obtained from three mice per group. Liver biopsies were briefly homogenized mechanically in PBS, filtered using a 100 *μ*m cell strainer (BD Biosciences, Franklin Lakes, NJ) and then placed in ACK lysing buffer (Lonza, Basel, Switzerland) to remove red blood cells. One million hepatocytes were washed once in PBS containing 0.5% bovine serum albumin (BSA) and were resuspended in 100 *μ*L of PBS/BSA buffer. Meanwhile, 1 × 10^6^ isolated cells were incubated with various conjugated monoclonal antibodies, including CD4-APC (1 : 400 dilution; BD Biosciences), CD3-FITC (1 : 100 dilution; BD Biosciences), CD8a-FITC (1 : 100 dilution; BD Biosciences), CD45R-PE (1 : 400 dilution; BD Biosciences), CD49b-APC (1 : 200 dilution; eBioscience, San Diego, CA), CD11c-PE (1 : 200 dilution; BD Biosciences), and F4/80-APC (1 : 100 dilution; eBioscience) for 20 min on ice, washed twice in PBS/BSA buffer, and re-suspended in 500 *μ*L of PBS/BSA buffer. Flow cytometric analysis was performed using a FACSCalibur apparatus (BD Biosciences) and the data were analyzed using WinMDI statistical software (Scripps, La Jolla, CA). Forward and side scatter parameters were used to gate on stained cells. 

### 2.8. Gel Electromobility Shift Assay

Gel electromobility shift assay (EMSA) was performed according to the manufacturer's recommendation (Promega, Madison, WI). The liver tissues were briefly homogenized in 200 *μ*L of solution A (10 mM HEPES (pH 7.9), 1.5 mM MgCl_2_, 10 mM KCl, 0.5 mM dithiothreitol, and 0.2 mM phenylmethylsulfonylfluoride), incubated on ice for 6 min, and then centrifuged at 6,000 rpm for 6 min. Pelleted nuclei were re-suspended in solution C (solution A supplemented with 420 mM NaCl and 20% glycerol) and incubated on ice with vigorous vortexing every 5 min for 20 min. The re-suspended pellet was centrifuged at 15,000 rpm for 15 min and the resulting nuclear extract supernatants were collected in a chilled Eppendorf tube. Consensus oligonucleotides were end-labeled using T4 polynucleotide kinase and [P^32^]-ATP for 10 min at 37°C. Gel shift reactions were assembled and incubated at room temperature. Subsequently, 1 *μ*L of gel loading buffer was added to each reaction and loaded onto a 6% nondenaturating gel. The gel was subjected to electrophoresis until the dye was four-fifths of the way down the gel. The gel was dried for 2 h at 80°C and exposed to film overnight at −70°C.

### 2.9. Histopathology and Immunohistochemistry

The liver tissues were fixed in 4% paraformaldehyde and cut into 30 *μ*m sections using a freezing microtome (Thermo Scientific, Germany). The sections were stained with hematoxylin and eosin (H&E) for pathological examination. For immunohistological staining, the liver sections were incubated in primary antibody against CD49 (1 : 5000 dilution; Santa Cruz Biotechnology), CD3 (1 : 1000 dilution; Abcam, Cambridge, UK), cytochrome P450 2E1 (1 : 2000 dilution; Abcam), and F4/80 (1 : 2000 dilution; Abcam). After rinsing in PBS, the sections were incubated in biotinylated secondary antibody. The tissue was incubated for 1 h in an avidin-peroxidase complex (ABC Elite Kit; Vector Laboratories, Burlingame, CA). After washing in PBS, the immunocomplex was visualized using 3,3-diaminobenzidine solution (2 mg/10 mL) containing 0.08% hydrogen peroxide in PBS. The sections were dehydrated in a series of graded alcohols, cleared in xylene, and coverslipped using Permount (Sigma-Aldrich). 

### 2.10. Statistical Analysis

The data were analyzed using the GraphPad Prism 6 software (version 6.00; GraphPad Software, San Diego, CA). Data are presented as mean ± SD. Group differences in APAP hepatotoxicity were analyzed using two-way ANOVA, with the factors being treatment and genotype, followed by Bonferroni's *post hoc* test. The rest were analyzed by the two-tailed Student's *t*-test. A value of *P* < 0.05 was considered statistically significant. Survival data were presented by Kaplan-Meier survival estimates and compared and calculated by the log-rank (Mantel-Cox) test in GraphPad Prism. All values are presented as mean ± SEM. Significance was set at *P* < 0.05 for all tests.

## 3. Results

### 3.1. Thiacremonone Reduced APAP-Induced Hepatotoxicity

**Figure 1:**
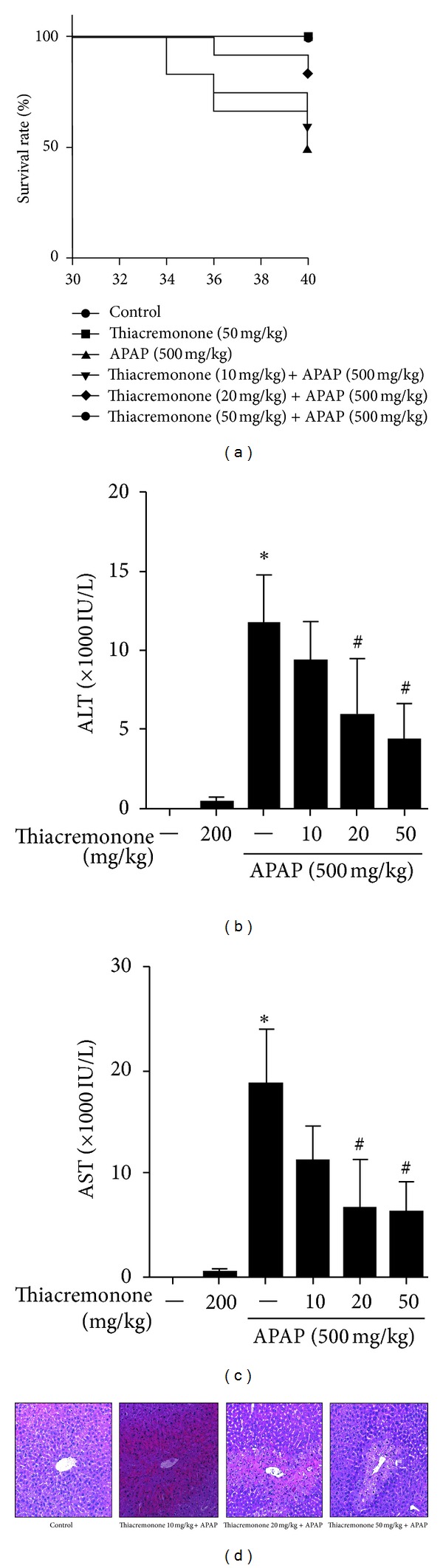
Inhibition of hepatotoxicity in thiacremonone pretreated mice. (a) The survival rates in control, APAP-treated, and thiacremonone pretreated mice were monitored for 40 h after 500 mg/kg APAP i.p. injection (*n* = 12). Thiacremonone pretreated mice demonstrated significantly higher survival rates compared to APAP-treated mice by the log-rank test. The levels of ALT (b) and AST (c) were determined in untreated controls and 40 h after i.p. injection with 500 mg/kg APAP. Mice were pretreated with thiacremonone (10, 20, and 50 mg/kg) for 7 days before APAP treatment. Thiacremonone pretreated mice had a significant reduction in the levels of ALT and AST compared to APAP-treated mice. (d) Liver sections were analyzed by H&E stain. Data are mean ± SD; ∗ indicates significant difference from control mice (*P* < 0.05); # indicates significant difference from APAP-treated mice (*P* < 0.05).

The effects of thiacremonone in drug-induced hepatotoxicity were investigated using APAP-treated mice. To assess survival, fasted mice were administered a single high dose of APAP (500 mg/kg) and were monitored for 40 h (Figure 1(a)). The survival rate of APAP-treated mice was significantly lower than that of control mice and thiacremonone-pretreated mice. The survival rate of APAP-treated mice was reduced to 50%, whereas the survival rate of 10, 20, and 50 mg/kg of thiacremonone pretreated mice was recovered to 58.3%, 83.3%, and 100%, respectively. At the end of the experiment, there was a significant difference in serum ALT and AST levels between control mice (8.6 and 3.0 IU/L) and APAP-treated mice (11754.8 and 18631.8 IU/L, *P* < 0.001), but the values were significantly decreased by 10 (9235.7 and 11063.8 IU/L), 20 (5797.8 and 6526.8 IU/L, *P* < 0.05), and 50 mg/kg (4264.8 and 6185.0 IU/L, *P* < 0.01) of thiacremonone in a dose-dependent manner (Figures 1(b) and 1(c)). Histopathological studies of mice liver tissues from the control group revealed normal hepatic cells with central vein and sinusoidal dilation. In the APAP-treated group, severe hepatotoxicity was observed in the form of severe necrosis along with the disappearance of nuclei. Histopathological analysis showed that the pathological lesions caused by APAP were very minimal in the groups pretreated with thiacremonone. Normal hepatocytes with regenerating hepatocytes and mild inflammation in the portal area were observed in the groups treated with thiacremonone (Figure 1(d)).

### 3.2. Thiacremonone Inhibits APAP-Induced Depletion of GSH, Overproduction of NO, and Lipid Peroxidation via Inhibition of Cytochrome P450 2E1 Expression

**Figure 2:**
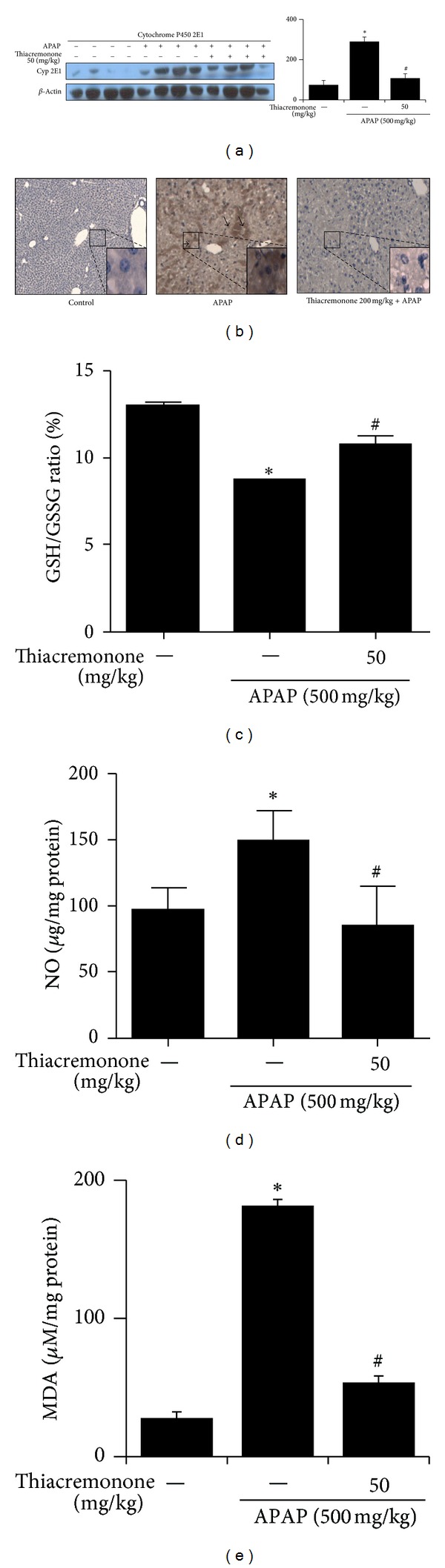
Decreased expression of cytochrome P450 2E1, GSH/GSSG ratio, NO, and lipid peroxidation in liver tissues of thiacremonone pretreated mice. (a) The expression of cytochrome P450 2E1, GSH/GSSG ratio, NO, and lipid peroxidation levels was determined in the total protein extracts of mice liver tissues by western blotting. (b) Immunohistochemical analysis of cytochrome P450 2E1 confirmed the intensities of staining for cytochrome P450 2E1 in the liver tissues of control, APAP-treated, and thiacremonone pretreated mice ((a) and (b)). (c) The hepatic NO in thiacremonone pretreated mice was significantly inhibited compared with that in APAP-treated mice. The expression and intensity of cytochrome P450 2E1 were decreased in the thiacremonone pretreated mice. (d) The hepatic GSH/GSSG ratio in thiacremonone pretreated mice was significantly increased compared with that in APAP-treated mice. (e) Hepatic lipid peroxidation in thiacremonone pretreated mice was significantly inhibited compared with that in APAP-treated mice. Data are mean ± SD; ∗ indicates significant difference from control mice (*P* < 0.05); # indicates significant difference from APAP-treated mice (*P* < 0.05).

Overexpression of cytochrome P450 2E1 plays a critical role in APAP-induced hepatotoxicity. To evaluate whether thiacremonone was related to the expression of cytochrome P450 2E1 in liver tissue, the protein expression of cytochrome P450 2E1 was determined by Western blotting and immunohistochemistry in liver tissue. The constitutive upregulation of cytochrome P450 2E1 expression by APAP injection was reduced in the thiacremonone pretreated mice liver tissue (Figure 2(a)). Immunohistochemistry also confirmed that the intensity of staining for cytochrome P450 2E1 was decreased in thiacremonone pretreated mice (Figure 2(b)). The metabolite of APAP could deplete GSH, causing lipid peroxidation and increasing NO generation. We investigated the difference in the GSH/GSSG ratio, NO production, and lipid peroxidation between APAP-treated mice and thiacremonone pretreated mice. The GSH/GSSH ratio was significantly decreased to about 67.4% in APAP-treated mice (*P* < 0.05) compared to control mice; however, the levels of GSH/GSSH ratio were significantly increased to about 123.6% in 50 mg/kg of thiacremonone pretreated mice (*P* < 0.05) compared to APAP-treated mice (Figure 2(c)). On the other hand, the levels of NO and lipid peroxidation were increased to about 156.5% and 661.5%, respectively, in APAP-treated mice (*P* < 0.05) compared to control mice. In mice pretreated with 50 mg/kg thiacremonone, the levels of NO and lipid peroxidation were significantly decreased (*P* < 0.05) to about 56.1% and 194.2%, respectively, compared to APAP-treated mice (Figures 2(d) and 2(e)).

### 3.3. Thiacremonone Inhibits APAP-Induced Overproduction of Proinflammatory Cytokine

**Figure 3:**
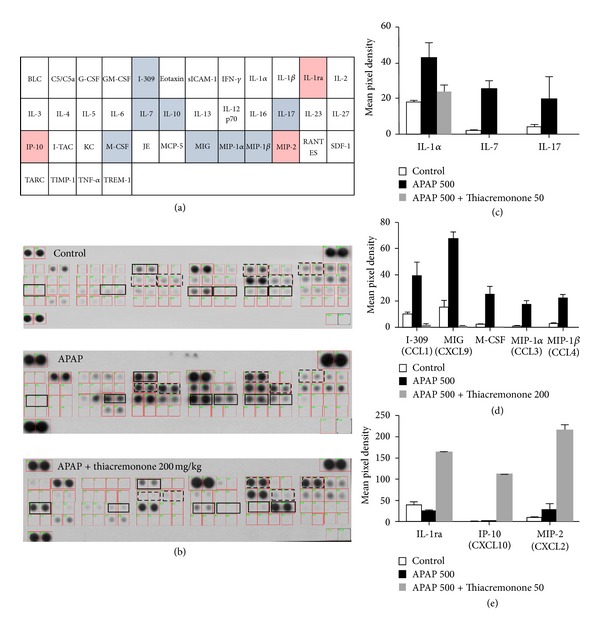
Downregulation of proinflammatory cytokines in the liver of thiacremonone pretreated mice. (a) Mouse cytokine array panel coordinates. Nitrocellulose membranes contain 40 different anticytokine antibodies printed in duplicate. (b) Mouse cytokine array panel indicates the cytokine expression difference in the liver tissues of control mice, APAP-treated mice, and thiacremonone pretreated mice. ((c), (d), and (e)) Representative blot from three independent experiments is shown. Positive controls show the manufacturer's internal positive control samples on the membrane. In particular, the expression levels of IL-1*α*, IL-7, IL-17, I-309, MIG, M-CSF, MIP-1*α*, and MIP-1*β* in thiacremonone pretreated mice were significantly decreased compared to APAP-treated mice.

To investigate the difference in the cytokine levels between APAP-treated liver and thiacremonone pretreated liver in mice, we conducted a cytokine array assay using a Mouse Proteome Array (Figures 3(a) and 3(b)). Among 40 tested cytokines, the levels of IL-1*α*, IL-7, IL-17, I-309 (chemokine C-C motif ligand 1, CCL1), M-CSF (macrophage colony-stimulating factor), MIG (C-X-C motif ligand 9, CXCL9), MIP-1*α* (CCL3), and MIP-1*β* (CCL4) were increased about 10-fold in APAP-treated mice in comparison to control mice (Figures 3(c) and 3(d)). However, these cytokines were significantly reduced in thiacremonone pretreated mice. In contrast, IL-1ra (an IL-1*α* receptor antagonist), IP-10, and MIP-2 were significantly elevated in thiacremonone pretreated liver tissues in comparison to control and APAP-treated livers (Figure 3(e)).

### 3.4. Decreased NF-*κ*B and STAT-1 Activity in Liver Tissue of Thiacremonone Pretreated Mice

**Figure 4:**
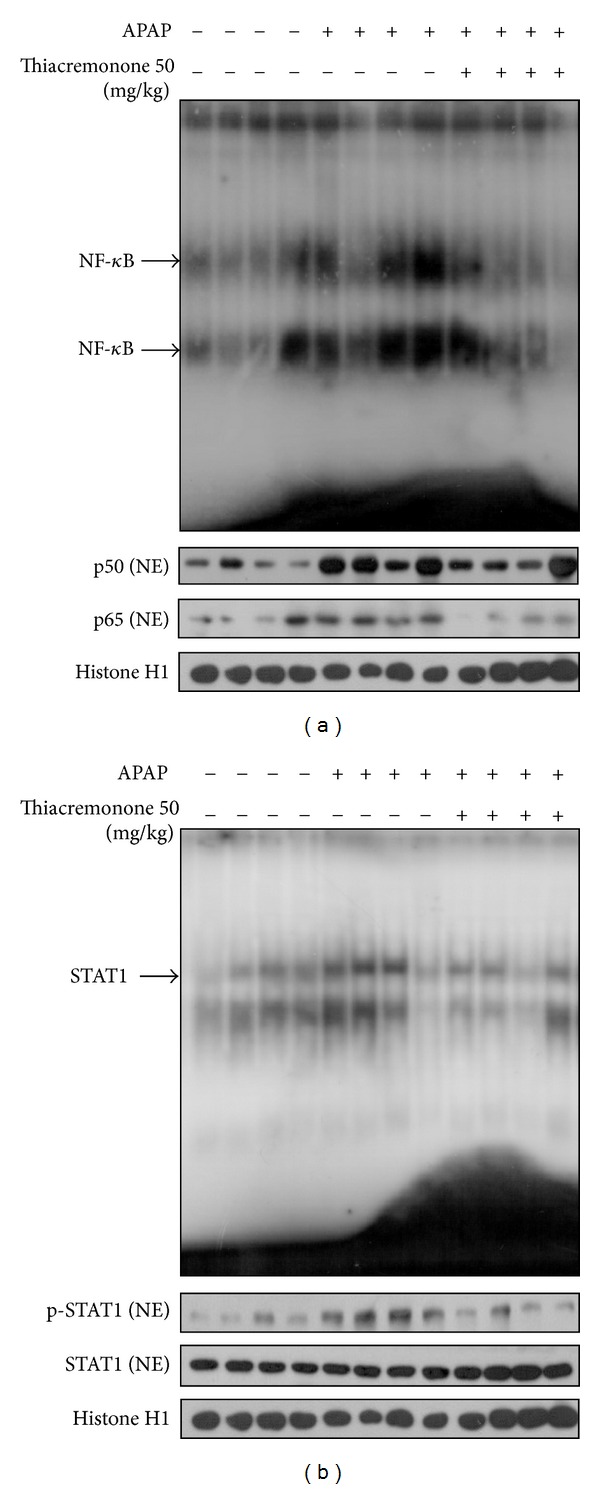
Reduction of NF-*κ*B and STAT1 in the liver of thiacremonone pretreated mice. (a) The expression of p50 and p65 phosphorylation in nuclear extracts (NE) was determined by Western blotting, and the DNA binding activity of NF-*κ*B was determined in the nuclear extracts of APAP-treated mice and thiacremonone pretreated mice liver tissues by EMSA. (b) The expression of p-STAT1 and STAT1 in NE was determined by Western blotting and the DNA binding activity of STAT1 was determined in the nuclear extracts of APAP-treated mice and thiacremonone pretreated mice liver tissues by EMSA.

To evaluate whether the hepatotoxicity was related with the inactivation of NF-*κ*B in the APAP-treated liver, the DNA-binding activity of NF-*κ*B was determined by EMSA in the liver tissue. Higher DNA-binding activity was found in the liver tissues of the APAP-treated liver compared with that of the thiacremonone pretreated liver. Moreover, inhibition of the translocation of p50 and p65 into the nucleus was also observed in the thiacremonone pretreated liver tissues (Figure 4(a)). We also evaluated whether the reduced hepatotoxicity in thiacremonone pretreated liver was related with the inactivation of STAT1 in the APAP-treated liver. DNA-binding activity of STAT1 was determined by EMSA in liver tissue. Higher DNA-binding activity was found in the liver tissues of the APAP-treated mice compared with that of the thiacremonone pretreated mice liver. Western blotting showed lower levels STAT1 and phosphorylation of STAT1 in the liver of the thiacremonone pretreated mice than those in the APAP-treated mice (Figure 4(b)).

### 3.5. Thiacremonone Reduces APAP-Induced Cytotoxic Immune Cell Infiltration

**Figure 5:**
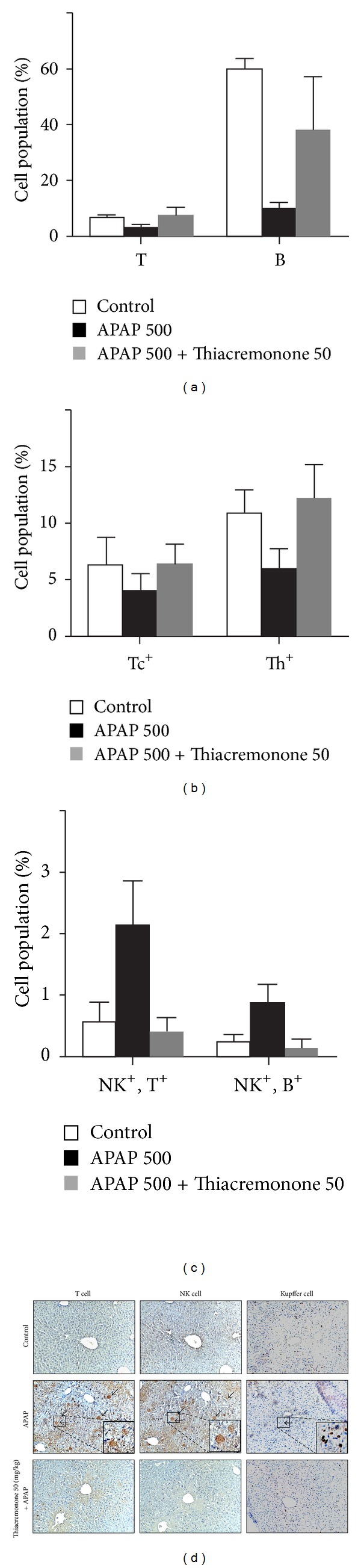
Reduction of the infiltration of immune cells and Kupffer cells into blood and liver tissues of thiacremonone pretreated mice. Blood ((a), (b), and (c)) and liver (d) tissues were separated at study termination. Flowcytometry analysis was performed using FACSCalibur flow cytometry. Representative data are shown. Data are mean ± SD of four experimental animals. (d) Photographs of the liver sections for Kupffer and NK cells after APAP in combination with vehicle or thiacremonone. Immunoreactivity with Kupffer and NK cells was expressed in the periportal (centrilobular) hepatocytes. APAP treatment exhibited the intense expression of Kupffer, NK, and Tc cells in the centrilobular sinusoids; however, pretreatment with thiacremonone caused mild staining for Kupffer and NK cells in the centrilobular sinusoids.

To investigate whether the inhibition of APAP-induced hepatotoxicity in thiacremonone pretreated mice was related to cytotoxic immune cell infiltration, we analyzed the distribution patterns of cytotoxic immune cells in liver tissues. Lymphocytes were isolated from liver tissues and were analyzed for cell phenotype by fluorescence activated cell sorting. The T, B, cytotoxic T (Tc), and helper T (Th) cell populations of APAP-treated mice were 3.1%, 10.1%, 4.1%, and 6.0%, respectively, compared to 6.7%, 59.4%, 6.3%, and 10.9%, respectively, of control mice. The values of thiacremonone pretreated mice were 7.6%, 38.3%, 6.4%, and 12.2%, respectively (Figures 5(a) and 5(b)). The double positive cells of NK and T and the double positive cells of NK and B cell population of APAP-treated mice liver were 2.1% and 0.9%, respectively, compared to 0.6% and 0.2%, respectively, of control mice. The values of thiacremonone pretreated mice were 0.4% and 0.1%, respectively (Figure 5(c)). These data suggest that promoting the infiltration of Th and NK, double positive cells of NK and T, and double positive NK and B cells could be reduced by thiacremonone. By staining the liver sections from APAP-treated mice, we found that there was a significant influx of Kupffer cells into the APAP-treated mice liver compared to control mice. The staining intensity showed a considerable decrease in the expression of Kupffer cells in thiacremonone treated liver tissues. The numbers and intensity of immune staining for NK cells and cytotoxic T cells were also higher in the livers of APAP-treated mice compared to the livers of control mice. However, the number and intensity of immunostaining for NK cells and cytotoxic T cells were decreased in the liver tissues of thiacremonone pretreated mice (Figure 5(d)). These data suggest that inhibition of infiltration of Kupffer cells, NK cells, and cytotoxic T cells could play a protective role in thiacremonone pretreated mice liver injury induced by APAP.

## 4. Discussion

In the present study, thiacremonone inhibited APAP-induced ALT, AST, and NO generation as well as lipid peroxidation accompanied by an increase of the GSH/GSSG ratio. Thiacremonone also decreased APAP-induced centrilobular hepatic necrosis and hepatic Kupffer cell, T cell, and NK cell infiltration as well as hepatic cytochrome P450 2E1 expression. The inhibitory effects of thiacremonone on proinflammatory cytokine expression were also found in the levels of I-309, IL-7, IL-10, IL-13, IL-17, M-CSF, MIG, MIP-1*α*, and MIP-1*β*. However, IL-1ra, IP-10, and MIP-2 were elevated by thiacremonone. These data suggest that thiacremonone could be a useful agent for the treatment of APAP-induced acute hepatic toxicity.

Excessive APAP in hepatocytes is metabolized into NAPQI mainly by cytochrome P450 2E1. Accumulated NAPQI kills hepatocytes by depletion of glutathione accompanied by oxidative stress [49]. NAPQI reacts with GSH, leading to its depletion by as much as 90%. Subsequently the metabolite covalently binds to hepatic cellular proteins to form 3-(cysteine-S-yl)-acetaminophen (APAP-Cys) adducts [1, 5, 6]. Peroxynitrite is then formed by a rapid reaction between NO and superoxide, and NO synthesis is increased by APAP treatment [50]. Peroxynitrite leads to nitration of tyrosine, which can attack a wide range of biological molecules such as lipids, proteins, and DNA. Peroxynitrite is normally detoxified by GSH/GSSG peroxidase, and GSH is depleted in APAP toxicity. Moreover, under the conditions of reduced cellular oxidant scavenging capability, peroxynitrite is highly toxic [51–53]. As a result, the normal detoxification mechanism for peroxynitrite is impaired and hepatocytes are damaged by oxidative stress [8]. Presently, the production of hepatic NO was decreased by treatment with thiacremonone. Thiacremonone also increased the GSH/GSSG ratio and decreased lipid peroxidation, indicating that thiacremonone could protect hepatocytes from oxidative cellular dysfunction. 

Elevated ALT and AST are characteristics of acute overdose of acetaminophen or other drugs or viral-induced causes of hepatic failure [54]. High serum ALT and AST levels in APAP-treated mice closely parallel histopathological liver damages, as previously reported in mouse models [1, 13, 55, 56]. Histopathological and serum analyses showed that pathological lesions caused by APAP were very minimal in livers of mice pretreated with thiacremonone. These data indicate that thiacremonone could be useful for prevention of liver damage by overdose of APAP.

Treatment of APAP could activate T cells, NK cells, and Kupffer cells. Activated T cells, NK cells, and Kupffer cells release numerous signaling molecules including hydrolytic enzymes, eicosanoids, nitric oxide, and superoxide. Kupffer cells may also release a number of pro-inflammatory cytokines including IL-1, IL-6, IL-7, IL-17, and TNF-*α* [13, 21, 57], which are markedly involved in the development of several autoimmune diseases such as rheumatoid arthritis, experimental autoimmune encephalitis, or Crohn's disease, as well as in the development of inflammatory responses [58]. Other cytokines such as I-309, M-CSF, MIG, MIP-1*α*, and MIP-1*β* belong to a family of inflammatory chemokines involved in the acute inflammatory state in the recruitment and activation of several immune cells [11, 12, 20]. These chemokines are reported to be secreted by damaged hepatocytes, T cells, NK cells, and Kupffer cells, which stimulates the attraction of NK cells, monocytes and a variety of other immune cells to the regions of hepatic necrosis [1, 5, 59, 60]. The expression levels of I-309, M-CSF, MIG, MIP-1*α*, MIP-1*β*, IL-7, IL-13, and IL-17 were increased following APAP pretreatment; however, these chemokine profiles were reduced by pretreatment with thiacremonone. Moreover, APAP-induced activation of Kupffer cells, cytotoxic T cells, and NK cells was reduced. Therefore, it is possible that thiacremonone could be hepatoprotective through reduced release of proinflammatory cytokines and chemokines accompanied with inactivation of Kupffer, NK, and Tc cells. Several chemokines, such as IP-10, IL-1ra, and MIP-2, can stimulate proliferation of hepatocytes and thus repair damaged liver [61]. Treatment with MIP-2 was more effective for the prevention of APAP-damaged liver injury. In addition, MIP-2 can maintain hepatocyte proliferation in cells exposed to APAP [21, 62]. IP-10 may be able to induce the hepatocyte growth factor as a mitogen. IP-10 has protective effects in APAP-induced hepatotoxicity, and these protective effects are associated with the induction of the MIP-2 receptor on hepatocytes [11, 12, 24]. APAP-induced hepatic necrosis was either reduced or resolved by IL-1ra. Administration of IL-1ra and lowering of the serum levels of IL-1*α* can bestow a partial hepatoprotective effect [15, 55, 63]. These chemokines could be released in damaged NK, T cells, and Kupffer cells. The damage of hepatic Kupffer cells, T cells, and NK cells was decreased by pretreatment with thiacremonone. We also discovered that thiacremonone increased MIP-2 and IP-10, the two factors responsible for hepatocyte regeneration. Thus, increasing the effect on these chemokines by thiacremonone could also result in direct recovery of hepatotoxicity or in indirect reduction of damaged Kupffer, T cells, and NK cells, thus reducing hepatotoxicity. 

NF-*κ*B translocation to the nucleus is required for the induction of a number of cytokines that are involved in APAP-induced hepatotoxicity [64]. Inflammatory stimuli, such as TNF-*α* and IL-1**β**, are activated by the ubiquitous NF-*κ*B pathway. Engagement of the TNF receptor (TNFR) and IL-1**β** receptor (IL-1*β*R) causes activation of NF-*κ*B [65]. NF-*κ*B regulates the expression of genes that control inflammatory mediators including nitric oxide synthase II (NOS II), TNF-*α*, IL-1*β*, and cyclooxygenase-2, each of which has been implicated in hepatotoxicity [66]. Substances such as curcumin, anetholdithiolthione, and silymarin protect the liver against damage via inhibition of NF-*κ*B [67–69]. Preventing NF-*κ*B inhibits activated Kupffer cells from producing TNF-*α* [70]. Thus, inhibition of NF-*κ*B causes the decline of inflammatory mediator-dependent liver injury. A previous study reported the time dependent upregulation of STAT-1 in hepatocytes treated with APAP [28]. IFN-*γ* and TNF-*α* activate STAT1 and cause apoptosis of hepatocytes. A recent study demonstrated that NK and NK T cells produce IFN-*γ* and play a critical role in APAP-induced liver injury [11]. IFN-*γ* activates STAT1, which initiates inflammatory signals in APAP-induced hepatitis by the induction of multiple cytokines, chemokines, adhesion molecules, and Fas/FasL. TNF-*α* also activates STAT1 and STAT1 null cells are resistant to apoptosis by TNF-*α* [12, 27, 71]. Thus, NF-*κ*B and STAT1 activation in APAP treated hepatocytes appears to be the responsible factor for liver injury [72]. NF-*κ*B and STAT1 activation was increased in APAP-treated liver; however, downregulation of both NF-*κ*B and STAT1 was observed in mice pretreated with thiacremonone. These data indicate that inhibition of both STAT1 and NF-*κ*B pathways is linked to the preventive effects of thiacremonone on APAP-induced toxicity. Taken together, the present study indicates that thiacremonone could be a useful agent for the treatment of overdose APAP-induced hepatic failure.
